# Architects of Aggression: The Molecular Blueprint of Glioma Progression

**DOI:** 10.3390/genes17091120

**Published:** 2026-09-15

**Authors:** David Aebisher, Jakub Tylutki, Angelika Myśliwiec, Nazarii Kozak, Dorota Bartusik-Aebisher

**Affiliations:** 1 Department of Photomedicine and Physical Chemistry, Faculty of Medicine, University of Rzeszów, 35-310 Reszów, Poland; 2 English Division Science Club, Faculty of Medicine, University of Rzeszów, 35-310 Rzeszów, Poland; jt130173@stud.ur.edu.pl (J.T.); nk130099@stud.ur.edu.pl (N.K.); 3 Department of Biochemistry and General Chemistry, Faculty of Medicine, University of Rzeszów, 35-310 Reszów, Poland; amysliwiec@ur.edu.pl

**Keywords:** glioblastoma, cellular plasticity, G-CIMP transition, synaptic hijacking, clonal evolution, metabolic reprogramming

## Abstract

Despite decades of clinical validation, glioblastoma (GBM) treatment remains tethered to a dismal 15-to-16-month survival plateau, heavily thwarted by the ys blood–brain barrier (BBB) and profound cellular heterogeneity. While the 2021 World Health Organization Classification of Tumors of the Central Nervous System (WHO CNS5) fundamentally reoriented diagnosis around definitive molecular signatures, such as isocitrate dehydrogenase (IDH)-wildtype status, epidermal growth factor receptor (EGFR) amplification, and +7/−10 chromosomal alterations, the ultimate obstacle to clinical efficacy is the tumor’s intense non-genetic plasticity. Malignant cells reject rigid hierarchies; single-nucleus insights reveal a fluid transcriptomic continuum spanning neurodevelopmental lineages, novel glia-like or neuronal-like states, and highly resilient proneural-mesenchymal (PM) hybrid populations. This intrinsic dynamism is reinforced by functional neuro-gliomal integration into the host brain via electrochemical TM synapses, an immunologically cold niche dominated by secreted phosphoprotein 1 (SPP1+) myeloid cells, and a self-reinforcing hypoxic-angiogenic loop. Under cytotoxic therapy, these networks execute rapid adaptive remodeling, selecting for hypermutator phenotypes and specialized senescence-associated secretory phenotypes (SASP) that dictate aggressive recurrence.

## 1. Introduction

Despite decades of scientific investigation, glioblastoma (GBM) remains the most devastating primary brain tumor [1,2]. The clinical standard of care, which includes maximal surgical cytoreduction paired with concurrent radiotherapy and alkylating chemotherapy using temozolomide (TMZ), has remained virtually unchanged for over two decades now [3,4]. The median overall survival (OS) for diagnosed patients remains constrained to 15–16 months, with five-year survival rates persisting below 7% [2,5,6]. This stagnation does not reflect therapeutic efficacy; rather, it highlights the profound limitations of current protocols and the formidable obstacles hindering durable interventions.

The durability of this therapeutic plateau stems from a bipartite obstruction involving both anatomical resistance and intrinsic cellular dynamism. While the selective permeability of the blood–brain barrier (BBB) and active efflux transport systems aggressively deplete intracranial drug concentrations, a major driver of treatment resistance is the tumor’s profound cellular heterogeneity [1,6,7,8,9]. GBM evades targeted intervention by eschewing a static architectural state, functioning instead as a non-genetically plastic, interconnected cellular network that executes rapid, adaptive remodeling under cytotoxic pressure [10].

To capture this biological complexity, the fifth edition of the World Health Organization Classification of Tumors of the Central Nervous System (WHO CNS5) introduced a revised taxonomic framework in 2021 [11,12]. By abandoning subjective histopathology, the classification now relies on an integrated, molecularly driven grading framework, where diffuse gliomas are now strictly divided into adult-type and pediatric-type categories [13]. Within the pediatric-type category, diffuse low-grade gliomas constitute a distinct group of molecularly defined entities that should be distinguished from adult-type diffuse gliomas despite potential histopathological similarities. This distinction reflects the integrated molecular framework introduced by the WHO CNS5 classification and emphasizes the importance of age-associated and molecular criteria in contemporary glioma diagnostics. For adult patients, the classification is streamlined into three distinct groups based on isocitrate dehydrogenase (IDH) mutation status and chromosomal 1p/19q codeletion: astrocytoma, IDH-mutant; oligodendroglioma, IDH-mutant and 1p/19q-codeleted; and GBM, IDH-wildtype [14]. While this review primarily focuses on IDH-wildtype GBM as the archetypal paradigm of lethal malignancy, selected high-grade entities, including IDH-mutant astrocytomas undergoing malignant progression and high-grade pediatric diffuse midline and hemispheric gliomas, are discussed to illustrate convergent evolutionary trajectories, shared epigenetic reprogramming, and common microenvironmental dependencies.

Crucially, the diagnosis of GBM is now strictly confined to adult, IDH-wildtype malignancies [15]. Histopathological features are no longer considered in isolation, as an adult infiltrating astrocytic glioma may be diagnosed as glioblastoma, IDH-wildtype, CNS WHO grade 4, if it exhibits specific molecular signatures, namely TERT promoter mutation, EGFR amplification, or combined whole-chromosome 7 gain and chromosome 10 loss (+7/−10) [15,16]. This reclassification applies even in the total absence of classic microscopic hallmarks like necrosis or microvascular proliferation [11]. Ultimately, this paradigm shift ensures that diagnostic taxonomies mirror intrinsic biological behaviour over mere morphology, thereby aligning therapeutic clinical trials with true genetic reality.

GBM cells do not conform to a rigid, unidirectional hierarchy [17], as single-cell RNA sequencing reveals that malignant cells transition across a fluid continuum of four principal states that recapitulate normal neurodevelopmental lineages: astrocyte-like (AC-like), mesenchymal-like (MES-like), oligodendrocyte progenitor-like (OPC-like), and neural progenitor-like (NPC-like) states [17,18,19]. These states are influenced by distinct microanatomical niches. The perivascular niche, governed by NOTCH1 signaling, sustains the stem-like, therapeutic-resistant OPC-like and MES-like subpopulations [20]. In contrast, the hypoxic and necrotic cores harbor quiescent, slow-cycling cells in the hypoxia-dependent MES-like state, protecting them from therapies that target active proliferation [19,21].

This transcriptomic flexibility represents a highly coordinated mechanism of therapeutic escape. For example, continuous pharmacological targeting of EGFR does not typically result in target mutation or reactivation. Instead, the tumor cells adaptively reprogram their transcriptomic and proteomic profiles, rewiring growth signals to bypass EGFR blockades through the upregulation of platelet-derived growth factor receptor alpha (PDGFRA) signaling, ultimately allowing GBM to easily circumvent therapies designed to target single molecular pathways [22,23,24,25].

Remarkably, gliomas do not exist as isolated cellular islands within the brain parenchyma. Activity of the nervous system has been identified as a major driver of progression in many primary brain cancers [26,27,28]. GBM cells functionally and electrically integrate into neural circuits by forming bona fide glutamatergic synapses with host neurons. Through calcium-permeable α-amino-3-hydroxy-5-methyl-4-isoxazolepropionic acid receptors (AMPARs) localized on specialized tumor microtubes (TMs), malignant cells hijack physiological neuronal activity to stimulate tumor progression. In certain high-grade tumors, altered expression of chloride cotransporters, such as overexpressed sodium-potassium-chloride cotransporter 1 (NKCC1) and downregulated potassium-chloride cotransporter 2 (KCC2), even converts traditionally inhibitory gamma-aminobutyric acid (GABA)ergic signaling into a growth-promoting, depolarizing stimulus [29,30,31,32,33,34]. This active integration establishes a positive feedback loop, where tumor cells promote regional hyperexcitability, which in turn fuels activity-dependent tumor progression, invasion, and therapy resistance.

To ensure a rigorous and balanced synthesis, a comprehensive literature search was conducted across the PubMed/MEDLINE, Web of Science, Scopus, and Google Scholar databases covering publications from inception through early 2026, with an emphasis placed on landmark translational breakthroughs and clinical trials published between 2015 and 2026. Eligible studies included peer-reviewed original research articles, high-impact reviews, and clinical trial reports published in English. Articles were selected based on mechanistic depth and relevance to molecular progression. Given the narrative nature of this review, the literature selection process may be subject to selection bias. Furthermore, no formal quantitative evidence synthesis or meta-analysis was performed.

## 2. Materials and Methods

### 2.1. Review Design and Literature Search

This review was conducted as a narrative literature review focusing on the molecular mechanisms underlying glioma initiation, progression, cellular plasticity, metabolic adaptation, tumor–microenvironment interactions, and treatment resistance. The literature search was designed to integrate evidence from genomic, epigenomic, transcriptomic, metabolic, and microenvironmental studies of glioma, with particular emphasis on glioblastoma (GBM) and molecularly defined high-grade glioma entities.

Relevant publications were identified through searches of PubMed and Google Scholar. The search strategy combined terms related to glioma biology and disease progression, including glioblastoma, high-grade glioma, cellular plasticity, clonal evolution, epigenetic remodeling, G-CIMP, IDH, H3K27M, H3 G34, metabolism, tumor microenvironment, glioma stem cells, synaptic integration, hypoxia, angiogenesis, immunosuppression, temozolomide resistance, and hypermutation. Additional relevant studies were identified by screening the reference lists of key publications.

### 2.2. Study Selection and Eligibility Criteria

Studies were selected based on their relevance to the molecular and cellular mechanisms discussed in this review. Priority was given to peer-reviewed original research articles, systematic and narrative reviews, genomic and transcriptomic studies, and clinically relevant investigations that provided mechanistic or translational evidence.

Particular emphasis was placed on studies investigating: (i) molecular alterations defining glioma initiation and progression; (ii) epigenetic and chromatin remodeling mechanisms; (iii) metabolic plasticity and nutrient adaptation; (iv) interactions between malignant cells and the neural and immune microenvironment; (v) glioma stem-cell niches and cellular state transitions; and (vi) treatment-induced clonal evolution and mechanisms of therapeutic resistance.

Studies were excluded when they were unrelated to glioma biology, lacked sufficient mechanistic or clinical relevance to the topics addressed, or did not provide information contributing to the molecular framework of glioma progression. Because this article represents a narrative review rather than a systematic review or meta-analysis, no quantitative pooling of effect estimates was performed.

### 2.3. Data Extraction and Thematic Synthesis

Information extracted from the selected literature included molecular alterations, affected signaling pathways, cellular phenotypes, epigenetic mechanisms, metabolic adaptations, microenvironmental interactions, experimental models, and reported consequences for tumor progression or therapeutic resistance.

The evidence was organized into six interconnected thematic domains: (1) genomic foundations and initiating events; (2) epigenetic architecture and chromatin remodeling; (3) metabolic reprogramming and bioenergetic plasticity; (4) tumor–microenvironment interactions, including neuronal and myeloid signaling; (5) clonal evolution and cellular plasticity; and (6) treatment-induced evolutionary bottlenecks and recurrence.

Particular attention was given to studies employing contemporary molecular approaches, including single-cell and single-nucleus transcriptomics, longitudinal tumor profiling, genomic sequencing, epigenomic analyses, and functional experimental models. Findings from these studies were integrated to construct a mechanistic framework linking genetic alterations with epigenetic states, metabolic adaptation, microenvironmental signaling, cellular-state transitions, and treatment resistance.

## 3. Results

### 3.1. The Genomic Foundation

At the core of this neoplastic ontogeny lie three interconnected, non-redundant signaling networks: the receptor tyrosine kinase (RTK)/Ras/phosphatidylinositol 3-kinase (PI3K) axis, the p53 tumor suppressor pathway, and the retinoblastoma (Rb) cell cycle checkpoint [35,36,37,38]. Comprehensive genomic sequencing from The Cancer Genome Atlas (TCGA) consortium established that approximately 80% to 90% of GBMs harbor at least one genomic alteration across these core axes, collectively enabling cells to evade growth suppression, sustain proliferative signalling, and resist apoptosis [39,40].

The RTK/Ras/PI3K pathway functions as the primary metabolic and proliferative engine of the tumor, transducing extracellular mitogenic signals into sustained intracellular growth commands. EGFR alterations represent the most frequent aberration in GBM, occurring in approximately 50% of cases through focal gene amplifications, point mutations, or intragenic rearrangements, yet half of them are in-frame deletions [39,41]. It is also worth noting that the missense mutations are rather mutually exclusive with the in-frame deletions [42]. Chief among these structural alterations is the EGFRvIII mutant, an in-frame deletion of exons 2 through 7 that results in the loss of the extracellular ligand-binding domain, causing constant, ligand-independent autophosphorylation of the intracellular tyrosine kinase domain. In other words, it leads to constitutively active mutant receptors [43,44]. This persistent activation recruits the p85 regulatory subunit of PI3K, which subsequently triggers the downstream AKT/mTOR cascade, driving protein synthesis and metabolic adaptation [45]. Despite being an attractive, tumor-specific neoantigen, therapeutic strategies targeting EGFRvIII, including the peptide vaccine rindopepimut (CDX-110), EGFR-directed tyrosine kinase inhibitors (TKIs), and third-generation CAR-T cells, have largely failed to improve overall survival in phase III trials [6,36]. These translational setbacks are predominantly driven by profound intratumoral heterogeneity, poor drug penetration across the BBB, rapid target antigen loss under selective immune pressure, and compensatory hyperactivation of redundant RTK networks, notably PDGFRA and MET [6,23,42].

Alternatively, PDGFRA amplification and mutation, which occur in approximately 10% of GBMs, serve as a parallel, mutually exclusive node of RTK activation. They are particularly prevalent in the proneural transcriptomic subtype, occurring in a third of these tumors [35,36,39,46,47,48]. This hyperactive RTK network is further unleashed by the genetic deletion or mutational inactivation of negative regulators, most notably the dual-specificity phosphatase and tensin homolog (PTEN), which normally antagonizes PI3K by dephosphorylating phosphatidylinositol (3,4,5)-trisphosphate (PIP_3_) [49].

Concurrently, cellular safety mechanisms designed to halt the cell cycle or trigger apoptosis are systematically dismantled. The p53 pathway, which orchestrates cellular responses to DNA damage, hypoxia, and oncogenic stress, is inactivated in the vast majority of cases [38,50,51]. While direct inactivating mutations in the DNA-binding domain of TP53 are exceedingly common, acting as early initiating events in up to 67% of secondary GBMs, alternative mechanisms of pathway suppression are frequently utilized [51,52]. These include focal amplifications of the oncogenic ubiquitin ligases MDM2 and MDM4, which target p53 for proteasomal degradation, as well as homozygous deletions of the chromosomal locus 9p21.3 [38,53]. This single locus contains the CDKN2A gene, which uniquely utilizes alternative reading frames to encode both p16^INK4A^ and p14^ARF^ [52,54,55]. Because p14^ARF^ directly binds and inhibits MDM2, its loss functionally mimics a direct TP53 mutation, allowing damaged cells to bypass apoptosis and accumulate additional driver mutations [55]. Interestingly, direct TP53 mutations predominate in the proneural subtype (~54%) but are virtually absent in classical GBMs, which rely almost exclusively on upstream MDM2/4 amplification or CDKN2A loss to silence the axis [25,56].

Parallel to p53 inactivation, cell cycle checkpoint regulation is compromised via the Rb pathway [35]. In a healthy physiological context, the retinoblastoma protein (pRb) complexes with and suppresses E2F transcription factors, preventing the transcription of genes required for G1-to-S phase transition. Upon mitogenic activation, cyclin-dependent kinases 4 and 6 (CDK4/6), complexed with Cyclin D, phosphorylate pRb, thereby releasing E2F and promoting cell cycle progression [35,53,57,58]. In GBM, this restrictive checkpoint is compromised by homozygous deletion of the cyclin-dependent kinase inhibitor CDKN2A. This locus encodes the CDK4/6 inhibitor p16^INK4A^ [59], a critical suppressor that restrains proliferation and curbs VEGF-driven angiogenesis [60,61]. This deletion, often co-occurring with focal amplification of CDK4 or CDK6 and inactivating mutations of the RB1 gene itself, ensures that the G1 checkpoint is permanently deactivated, fueling the rapid, exponential clonal expansions characteristic of these aggressive malignancies [35].

#### Core Pathways and Initiation Events

Historically, the clinical transition from low-grade (WHO grade 2) diffuse astrocytomas to secondary GBM was defined purely by morphological criteria, including the development of microvascular endothelial proliferation and palisading necrosis [51,62,63]. However, the modern molecular framework recognizes that these macroscopic changes are driven by distinct, predictable genetic bottlenecks, most notably the loss of heterozygosity (LOH) on chromosome 10q and the homozygous deletion of CDKN2A/B [51,62,64].

The CDKN2A/B deletion frequently encompasses the adjacent MTAP gene, which encodes methylthioadenosine phosphorylase, which is a key enzyme in the salvage pathway for purine and methionine biosynthesis [54,65,66]. Because of this tight physical proximity, heterozygous or homozygous deletions of MTAP occur concurrently with CDKN2A/B loss in up to 99.5% of adult-type diffuse gliomas [66]. This concurrent loss has emerged as a major diagnostic opportunity, where immunohistochemical (IHC) staining for MTAP acts as a highly sensitive (~92.3%) and specific (~97.5%) surrogate marker for identifying CDKN2A/B homozygous deletion in laboratories where advanced next-generation sequencing or fluorescence in situ hybridization (FISH) is cost-prohibitive [54,65,66,67].

The malignant progression of diffuse gliomas represents a classical Darwinian model of clonal evolution, characterized by sequential selective pressures, spatial bottlenecking, and the accumulation of synergistic genetic aberrations [68]. According to algorithmic modeling, alterations in IDH1, phosphatidylinositol-4,5-bisphosphate 3-kinase catalytic subunit alpha (PIK3CA), and ATRX serve as foundational, early-stage events in tumor progression, whereas aberrations involving PTEN, neurofibromatosis type 1 (NF1), and EGFR typically emerge during later stages of clonal evolution [68]. A parallel evolutionary trajectory is documented in lower-grade gliomas, where trunk mutations in IDH1, TP53, and ATRX manifest during initial tumorigenesis and remain stably conserved as the disease advances from the primary diagnosis to subsequent relapse [69,70].

The evolutionary synergy of these sequential mutations is also demonstrated by functional models in which dual inactivation of p53 and PTEN in neural stem cells (NSCs) induces a highly penetrant, acute-onset high-grade phenotype that closely mirrors the clinical, pathological, and transcriptomic behavior of human GBM [44,71]. Both the p53 and PTEN/PI3K axes converge on the regulation of c-MYC; p53 directly binds the c-MYC promoter to repress its transcription [72], whereas downstream PI3K signaling modulates c-MYC translation and proteasomal degradation [73]. Ultimately, this dual loss arrests cellular differentiation, preserves a stem-like state with high self-renewal capacity, and actively stabilizes the MYC proto-oncogene to drive continuous cell division [71,74,75,76]. During this transition, cells experience a dramatic metabolic shift: as the epigenome undergoes global demethylation from a restricted CpG island (CGIs) methylator phenotype (G-CIMP)-high to a more transcriptionally permissive G-CIMP-low state, genes encoding key glycolytic enzymes, such as lactate dehydrogenase A (LDHA), are selectively upregulated, driving a metabolic transition toward the classic glycolytic Warburg phenotype [77] (Table 1).

### 3.2. Epigenetic Architecture & Chromatin Remodeling

#### 3.2.1. The G-CIMP Phenotype

The epigenomic landscape of IDH-mutant gliomas is fundamentally sculpted by the glioma G-CIMP [84]. Somatic point mutations in IDH1 or IDH2, most frequently occurring as the IDH1 R132H substitution, confer a neomorphic enzymatic activity that catalyzes the reduction of α-ketoglutarate to the oncometabolite D-2-hydroxyglutarate (2-HG) [84,87,88,89]. This structural analogue competitively inhibits α-ketoglutarate-dependent dioxygenases, including the ten-eleven translocation (TET) family of 5′-methylcytosine hydroxylases, which normally initiate active DNA demethylation. The resulting epigenetic block establishes a concerted, genome-wide hypermethylation at CpG-rich promoter regions [90,91,92,93,94].

Within adult diffuse gliomas, this phenotype exhibits significant intra-subtype heterogeneity. A supervised random forest analysis of nearly 200 longitudinal glioma fragments revealed that G-CIMP-positive tumors partition into distinct epigenetic states: 67.4% present as G-CIMP-high at primary diagnosis, whereas 5.5% manifest the highly aggressive G-CIMP-low phenotype [95]. While the G-CIMP-high state correlates with favorable clinical outcomes (median survival ~7.2 years), primary or recurrent G-CIMP-low tumors are associated with a dismal prognosis (median survival ~2.7 years), mimicking the clinical aggressiveness and stem cell-like transcriptomic profile of IDH-wildtype GBMs [41,84,96].

The transition from G-CIMP-high to G-CIMP-low at tumor recurrence constitutes a major molecular bottleneck, driven by extensive DNA demethylation. Approximately 81.58% (558 of 684) of the hypomethylated CpG sites associated with this shift reside within intergenic “open sea” regions, particularly affecting functional tissue enhancers. This selective demethylation exposes DNA binding motifs recognized by the oncogenic transcription factors STAT3 and c-JUN/AP-1. Specifically, 5.12% of these hypomethylated enhancer regions share a dual 5′-TGA{G/C}TCA-3′ (AP-1) and 5′-TTGT-3′ (SOX) motif signature, driving plastic cellular states and tumor cell stemness [95,96].

This progressive epigenomic remodeling is also characterized by a seven-CpG hypomethylated biomarker signature in primary G-CIMP-high tumors that accurately predicts future malignant transformation to WHO grade IV G-CIMP-low recurrences [97]. Clinically, G-CIMP-low tumors display a high rate of homozygous deletion of the CDKN2A/B locus (38.9% in G-CIMP-low vs. 5.1% in G-CIMP-high), a molecular alteration that immediately upgrades any IDH-mutant astrocytoma to WHO Grade IV [94]. Although newly diagnosed IDH-mutant lower-grade gliomas show therapeutic vulnerability to the dual mIDH1/2 inhibitor vorasidenib as demonstrated in the INDIGO clinical trial [79], the emergence of contrast enhancement during progression frequently correlates with G-CIMP-low epigenomic transitioning and clinical resistance.

The pathophysiological consequences of G-CIMP-associated hypermethylation extend beyond local gene silencing to the complete collapse of three-dimensional (3D) chromatin architecture. In normal glial cells, topologically associated domains (TADs) are strictly partitioned by the CCCTC-binding factor (CTCF), a methylation-sensitive zinc-finger insulator protein that forms structural loops at domain boundaries to restrict enhancer-promoter interactions [98,99,100]. Under the hypermethylated G-CIMP-high state, the CTCF consensus binding motifs become highly methylated. This stereochemical alteration prevents CTCF from binding, leading to a catastrophic loss of insulation between adjacent topological domains [101,102].

Specifically, at the chromosomal boundary flanking the PDGFRA oncogene, CTCF depletion permits a strong, constitutive enhancer within the neighboring FIP1L1 house-keeping gene domain to physically interact with the PDGFRA promoter. This “enhancer hijacking” event induces robust, ligand-independent upregulation of PDGFRA, fueling glioma cell proliferation and signaling [102,103]. Exposure of IDH-mutant gliomaspheres to the DNA methyltransferase inhibitor 5-azacytidine restores CTCF binding at the FIP1L1-PDGFRA boundary and downregulates PDGFRA expression [103]. Conversely, CRISPR-Cas9-mediated disruption of the CTCF boundary motif in IDH-wildtype cells recapitulates this enhancer co-option and accelerates proliferation [102].

#### 3.2.2. Histone Variants

Although the WHO CNS5 classification strictly segregates pediatric-type from adult-type diffuse high-grade gliomas, pediatric high-grade entities provide indispensable mechanistic paradigms of oncohistone-mediated developmental arrest and chromatin deregulation that functionally parallel progression pathways in adult malignancies.

The fifth edition of the WHO Classification of Tumors of the Central Nervous System recognizes “Diffuse Midline Glioma (DMG), H3 K27-altered” as a distinct, uniformly lethal pediatric-type high-grade entity [104,105,106]. Approximately 80% of these aggressive brainstem and thalamic tumors are driven by a heterozygous, somatic missense mutation replacing lysine 27 with methionine (H3K27M) in either the non-canonical histone variant H3.3 (encoded by H3F3A) or canonical H3.1 variants (encoded by HIST1H3B/C) [104]. The substituted methionine side chain inserts directly into the catalytic SET domain of EZH2, the active methyltransferase subunit of the Polycomb Repressive Complex 2 (PRC2). This interaction inhibits PRC2 activity through competitive substrate blocking, resulting in a global, trans-reduction of the repressive epigenetic mark: trimethylation at lysine 27 (H3K27me3) across the entire genome [107,108].

This epigenetic repression, however, exhibits a distinct paradox: while global H3K27me3 is depleted, residual peaks of H3K27me3 are selectively retained at CGIs [109,110]. This non-uniform distribution is explained by an allosteric trapping mechanism [111]. PRC2 contains the EED subunit, which binds to pre-existing H3K27me3 marks, inducing an allosteric conformational shift that activates its methyltransferase subunit EZH2 to “write” downstream methylation [111,112]. The oncoprotein H3K27M preferentially binds to and selectively inhibits this allosterically activated form of PRC2. This high-affinity interaction selectively traps PRC2 at CGI recruitment hubs [113,114,115]. Because the complex is physically bound at these nucleation sites, it is incapable of lateral spreading, leading to localized transcriptional de-repression and the activation of developmental oncogenic programs [115].

An identical chromatin landscape is established in a subset of H3-wildtype DMGs and in posterior fossa ependymoma subtype A (EPN-PFA) through the aberrant re-expression of enhancer of zeste homologs inhibitory protein (EZHIP, also known as CXorf67) [116,117,118]. Normally restricted to the germline (oocytes and testes) to preserve totipotency and prevent premature gene silencing, EZHIP acts as a potent structural oncohistone mimic [112,119,120]. Its C-terminus encompasses a serine-rich sequence of amino acids that contain a highly conserved 13-amino-acid motif (K27M-like) with a critical methionine residue (M406) that mimics the H3K27M peptide sequence. EZHIP binds directly to the active site of EZH2, inhibiting its catalytic function and phenocopying the loss of genomic H3K27me3 spreading without requiring incorporation into the nucleosome [121].

In vivo models demonstrate that transgenic expression of EZHIP is biochemically more potent than H3K27M. In Drosophila melanogaster, ubiquitous expression of EZHIP induces larval lethality at the third-instar stage, whereas H3K27M allows progression to pupation. Correspondingly, while the tissue-specific expression of H3K27M impairs wing formation, EZHIP completely abolishes it [122].

The global loss of H3K27me3 allows the histone acetyltransferases p300 and CBP to deposit the active modification H3K27ac at newly accessible promoters [123]. This activates major oncogenic cascades, including the mitogen-activated protein kinase (MAPK) and PI3K/AKT/mTOR pathways, and upregulates MYC and EGFR [124,125,126]. This open chromatin conformation also impairs homologous recombination and base excision DNA repair pathways by transcriptionally silencing BRCA1 [127]. This repair defect renders H3K27M-mutant DMGs highly sensitive to the PARP inhibitor pamiparib in combination with radiotherapy [128].

Additionally, these tumors exhibit altered RNA epigenetics. They enrich m6A RNA methylation marks on cell cycle transcripts via the demethylase FTO and the reader YTHDF2 to promote translation [129]. This highly active cell cycle makes H3K27-altered DMGs selectively sensitive to aurora kinase inhibitors (AKIs), such as alisertib (AURKA inhibitor), barasertib (AURKB inhibitor), and AT9283 (pan-aurora kinase A/B/C inhibitor) [106].

#### 3.2.3. Epigenetic Rewiring of H3.3 G34R/V Gliomas

Diffuse hemispheric gliomas (DHG), H3 G34-mutant, represent 9% to 15% of pediatric and young adult hemispheric high-grade gliomas [130]. They occur predominantly in the temporo-parietal lobes at a median age of 15 years with median OS of around 18 months [131,132]. These aggressive malignancies almost invariably carry co-occurring somatic loss-of-function mutations in the chromatin remodeler ATRX (84%) and the tumor suppressor TP53 (95%) [133].

H3.3 G34R or the less frequent H3.3 G34V substitutions do not induce genome-wide changes in histone mark deposition. Instead, the amino acid replacement acts in cis conformation to disrupt H3K36me3 and H3K27me3 deposition, thereby impairing the recognition of the adjacent lysine 36 residue (an epigenetic mark essential for gene expression, transcription elongation, alternative splicing, and DNA repair) [134,135,136,137]. The bulky arginine or valine side chain directly blocks the binding of the histone reader ZMYND11, a transcriptional co-repressor that recognizes the transcription elongation mark H3K36me3. In normal development, ZMYND11 binds H3K36me3 to repress key forebrain progenitor regulator genes. In the presence of the H3.3 G34R mutation, this repression is lost, causing the persistent overexpression of the developmental regulators DMRTA2 and ARX [135,138,139].

Single-cell transcriptomic analyses have traced the cellular origin of H3.3 G34R/V gliomas to early cortical interneuron progenitors (CIPs) derived from ventral radial glial cells within the embryonic ganglionic eminences. These stalled progenitors express the homeobox transcription factors GSX2 and the DLX1/2 family. The H3.3 G34R mutation arrests these cells along this interneuronal development continuum, preventing terminal neuronal differentiation [140,141].

Because of this lineage-specific origin, these cells maintain a persistently open chromatin state at the active enhancer locus of GSX2. This spatial open conformation facilitates the formation of an ectopic chromatin loop that physically bridges the active GSX2 enhancer directly to the promoter of the nearby receptor tyrosine kinase PDGFRA, leading to excessive transcription of PDGFRA [141]. Under the selective pressure of TMZ chemotherapy, these tumors undergo clonal evolution, selecting for activating mutations or gene amplifications within the extracellular immunoglobulin-like domain of PDGFRA. This event overactivates the downstream MAPK-ERK pathway, promoting malignant glial expansion [133,142].

This lineage-specific arrest is also characterized by a complete lack of oligodendroglial programs, which are actively repressed by GSX2/DLX-mediated cell-fate specification [133]. Furthermore, H3.3 G34R/V expression impairs the recruitment of SETD2 and inhibits KDM4 demethylase activity, causing a localized loss of H3K36me3 and the transcriptional downregulation of the DNA repair enzyme O^6^-methylguanine-DNA methyltransferase (MGMT). This downregulation enhances these tumors’ sensitivity to alkylating agents like TMZ [137,143,144].

This repair defect also leads to genomic instability and the accumulation of cytosolic extrachromosomal DNA. This accumulation activates the cyclic GMP-AMP synthase (cGAS)/STING pathway and drives JAK/STAT signaling, transforming the tumor microenvironment (TME) into an immune-permissive niche that is highly responsive to immunostimulatory gene therapies [130,143].

#### 3.2.4. Post-Transcriptional Regulators of Chromatin Dynamics

Non-coding RNAs operate as structural scaffolds and post-transcriptional regulators, coordinating chromatin state transitions in GBM. The long non-coding RNA (lncRNA) HOTAIR (*HOX* transcript antisense intergenic RNA) is transcribed as a spliced, polyadenylated 2158-nucleotide transcript from the antisense strand of the HOXC locus on chromosome 12q13.13 [145,146]. HOTAIR is markedly upregulated in high-grade gliomas and serves as a physical scaffold for two major epigenetic silencing complexes. Its 5′ domain binds directly to the EZH2 catalytic subunit of PRC2 to drive H3K27 trimethylation, while its 3′ domain recruits the lysine-specific demethylase 1 (LSD1)-REST-CoREST1 complex, which demethylates activating H3K4me2 marks. This coordinated dual recruitment deposits repressive marks while erasing active transcription signals, silencing tumor suppressor genes and enhancing GBM cell invasion and therapeutic resistance [147,148,149].

Similarly, the lncRNA MALAT1 (Metastasis-Associated Lung Adenocarcinoma Transcript 1) localizes to nuclear speckles and physically binds to EZH2 and the chromatin-remodeling subunit BRG1, a core component of the SWI/SNF complex. This interaction recruits EZH2 to specific genomic loci, while altering alternative splicing and transcriptional programs to promote cell proliferation and suppress apoptosis [150,151,152]. Therapeutic resistance is also actively coordinated by SNHG12 (Small Nucleolar RNA Host Gene 12), which is epigenetically activated by DNA methylation at CpG islands within its promoter region in temozolomide-resistant GBMs, serving as a molecular shield that downregulates autophagy under genotoxic stress [150,153]. Furthermore, linc-RA1 (radioresistance-associated long intergenic non-coding RNA 1) physically binds to histone proteins to regulate H2B K120 monoubiquitination. This binding prevents histone deubiquitination by USP44, inhibiting autophagy and promoting radioresistance in vivo [154]. In contrast, the lncRNA AC016405.3 functions as a tumor suppressor. It is frequently downregulated in GBM, which relieves its sponging of miR-19a-5p and consequently leads to the downregulation of the methylcytosine dioxygenase TET2, promoting tumor cell survival [155]. These structural lncRNAs operate in tandem with oncogenic microRNAs (miRNAs) through competitive endogenous RNA (ceRNA) “sponging” networks to modulate critical signaling cascades [150,156]. For example, LINC00461 is highly overexpressed in glioma stem cells (GSCs). Upstream, HDAC6 binds the RNA-decay core subunit CNOT6 to stabilize LINC00461, which then sponges miR-485-3p. This sponging de-represses maternal embryonic leucine zipper kinase (MELK) and minichromosome maintenance protein 10 (MCM10), driving cell cycle progression [157]. Similarly, the lncRNA LPP-AS2 promotes tumorigenesis by sponging miR-7-5p, which de-represses the EGFR/PI3K/AKT/c-MYC pathway. FGD5-AS1 directly activates the Wntβ-catenin pathway to promote migration [158,159]. Exosomal Linc01060 regulates the MZF1/c-Myc/HIF1 axis to drive tumor progression under hypoxia, and FTX sponges miR-342-3p to enhance survival [160,161]. These lncRNA networks are balanced by specific oncogenic and tumor-suppressing miRNAs. The “oncomiR” miR-21 is frequently overexpressed in GBM, where it targets the tumor suppressors PTEN and RECK to promote stemness, proliferation, and invasion. Similarly, miR-10b facilitates parenchymal invasion by targeting HOXD10, which degrades extracellular matrix components [156,162]. Conversely, the tumor suppressor miR-34a, which regulates cell cycle arrest and apoptosis, is commonly lost [156]. Finally, the brain-enriched miR-181a targets the anti-apoptotic gene BCL-2 and components of the NF-κB signaling pathway. When its expression is restored, miR-181a inhibits DNA damage repair pathways, significantly sensitizing GBM cells to TMZ and radiotherapy [163,164].

### 3.3. The Metabolic Engine

#### 3.3.1. Bioenergetic Plasticity and the Glycolysis-OXPHOS Circuit

While historical paradigms classified GBM as an obligate glycolytic entity tethered exclusively to the Warburg effect, contemporary bioenergetic models reveal a highly plastic metabolic circuitry. Within this network, slow-cycling GSCs alternate fluidly between aerobic fermentation and mitochondrial oxidative phosphorylation (OXPHOS) to survive the highly variable, nutrient-restricted microenvironments of the core and the invasive margin [165,166,167]. Even in oxygen-abundant conditions, highly malignant glioma cells ferment the majority of their imported glucose into lactate, yielding only two moles of adenosine triphosphate (ATP) per mole of glucose [168,169]. This aerobic glycolysis is largely coordinated by hexokinase 2 (HK2), which catalyzes the committed initial phosphorylation step, and the dimeric isoform of pyruvate kinase M2 (PKM2), which creates an enzymatic bottleneck that shunts glycolytic intermediates toward macromolecular biosynthesis while accelerating lactate production [165,166,167].

Far from being a metabolic dead end, extracellular lactate acts as an autonomous paracrine and autocrine signaling ligand. It stimulates its cognate G-protein coupled receptor, hydroxycarboxylic acid receptor 1 (HCAR1/GPR81), to initiate a signaling cascade [170]. This receptor activation upregulates the monocarboxylate transporters MCT1 and MCT4, triggers mitochondrial biogenesis, and remodels epithelial-to-mesenchymal transition (EMT) proteins like E-cadherin and β-catenin [170,171].

This lactate efflux also establishes a complex metabolic symbiosis with the surrounding non-malignant brain architecture. Glioma-produced lactate is exported through MCT1 and MCT4 and selectively imported by adjacent glutamatergic neurons via MCT2, fueling their mitochondrial citric acid cycle [172,173,174]. By utilizing this tumor-derived monocarboxylate, adjacent neurons bypass their own rate-limiting glycolytic steps. This spares extracellular glucose for exclusive consumption by the glucose-addicted, highly proliferative glioma bulk [169]. To reinforce this loop, glioma cells secrete high levels of glutamate to excite glutamatergic neurons, driving them to consume more lactate and spare more glucose [175,176]. This metabolic agility bypasses monotherapeutic bottlenecks.

When microenvironmental glucose levels drop, the liver kinase B1 (LKB1) and AMP-activated protein kinase (AMPK) energy-sensing pathway coordinates a phenotypic pivot [177,178]. Under abundant nutrient conditions, highly elevated levels of miR-451 restrict the expression of CAB39 (MO25α), which is the critical scaffolding partner of the tumor suppressor LKB1, thereby keeping AMPK dormant and driving rapid mTORC1-dependent anabolic growth [178]. Nutrient deprivation downregulates miR-451, releasing CAB39 and enabling LKB1 to phosphorylate AMPK at Thr172 [178]. Activated AMPK restricts energy-intensive translation via the TSC2-Raptor axis while simultaneously initiating migratory and invasive programs that allow tumor cells to seek more favorable anatomical niches [177,178].

Furthermore, under localized serine and glycine starvation, reactive oxygen species (ROS) trigger AMPK activation, which stabilizes the transcriptional activity of HIF-1α [179]. This directly upregulates the de novo serine synthesis pathway (SSP) enzymes phosphoglycerate dehydrogenase (PHGDH), phosphoserine aminotransferase (PSAT1), and phosphoserine phosphatase (PSPH) to maintain survival [179]. Under glutamine deprivation, glioma cells also pivot to autophagy-mediated serine and glycine utilization rather than glycolytic intermediates to fuel one-carbon metabolic pathways, marked by the rapid activation of PSAT1, SHMT2, and MTHFD2 [180].

#### 3.3.2. Neuro-Chemical Scavenging

Glioma progression is similarly fueled by chemical scavenging of the brain’s abundant amino acid reservoir. While non-neoplastic astrocytes utilize GLT-1 (EAAT2) and GLAST (EAAT1) to clear extracellular glutamate and convert it to glutamine via glutamine synthetase (GS) [181,182,183], GBM cells upregulate specialized transporters including ASCT2 (SLC1A5), SNAT1, LAT1, and ATB0,+ (SLC6A14) to hyper-accumulate this circulating glutamine [184,185,186]. Mitochondrial glutaminase (specifically the kidney-type KGA and glutaminase C isoforms of GLS-1) then hydrolyzes this imported glutamine to yield glutamate and free ammonia, driving anaplerotic supply of α-ketoglutarate to the TCA cycle and supplying nitrogen for purine and pyrimidine biosynthesis [187]. Under low astrocytic EAAT1/2 transporter levels, GSCs become highly addicted to intracellular GLS activity to prevent a lethal amino acid deprivation response (AADR) [188].

To defend against mitochondrial ROS, GBM cells coordinate a protective antioxidant loop via upregulated system x_c_^−^, a heterodimeric transporter consisting of the functional light-chain xCT (SLC7A11) and heavy-chain chaperone CD98 (SLC3A2) [189,190]. By exporting intracellular glutamate in exchange for extracellular cystine, system x_c_^−^ provides the rate-limiting raw material for glutathione (GSH) synthesis, conferring robust resistance to genotoxic TMZ [189,190]. Crucially, this massive glutamate extrusion drives localized excitotoxicity, destroying adjacent neurons through N-methyl-D-aspartate receptors (NMDARs) and AMPARs, while autocrine binding to calcium-permeable AMPARs on the glioma cell membrane stimulates cell invasion and progression [190,191,192].

#### 3.3.3. Lipid Homeostasis

To satisfy the structural demands of cell division, GBMs execute a profound rewiring of lipid metabolism. EGFRvIII mutations orchestrate SREBP-1 activation, upregulating ATP-citrate lyase (ACLY), acetyl-CoA carboxylase (ACC), fatty acid synthase (FASN), and stearoyl-CoA desaturase 1 (SCD1) to drive autonomous de novo lipogenesis [193,194,195]. When glucose availability is restricted or under severe hypoxia, the tumor recruits acetyl-CoA synthetase 2 (ACSS2), directly transactivated by stabilized HIF-1α, to capture microenvironmental acetate as an alternative lipogenic precursor [196,197]. This enzyme additionally translocates to the nucleus to execute histone acetylation on crucial pro-survival and autophagy genes [196,198,199]. Phosphorylation of ACSS2 at Ser-267 by CDK5, fueled by O-GlcNAc transferase (OGT) activity, preserves ACSS2 from ubiquitin-mediated degradation, solidifying this bypass pathway [200].

Slow-cycling glioma subpopulations overexpress the lipid chaperone FABP7 to actively sequester polyunsaturated fatty acids (PUFAs), such as docosahexaenoic acid (DHA) and arachidonic acid (AA), directly from the brain parenchyma [201,202]. When bound to AA, FABP7 stimulates COX-2 and downstream prostaglandin E2 (PGE2) synthesis [202,203]. This PGE2 activates GSC self-renewal via the ARS2-MAGL signaling axis and polarizes tumor-associated macrophages (TAMs) into an immunosuppressive, IL-10-secreting phenotype, while monoacylglycerol lipase (MAGL) liberates free fatty acids from monoacylglycerols to support stemness [203].

Yet, this hyper-activated lipid uptake and synthesis risks lipid-induced ER stress and lipotoxicity. To circumvent this, glioma cells upregulate the endoplasmic reticulum-resident acyltransferases diacylglycerol acyltransferase 1 (DGAT1) and sterol O-acyltransferase 1 (SOAT1) to esterify excess free fatty acids and cholesterol into neutral triglycerides (TGs) and cholesteryl esters (CEs) sequestered within cytosolic lipid droplets (LDs) [194,204,205]. Pharmacological inhibition of SOAT1 floods the ER membrane with free cholesterol, activating a feedback mechanism that retains the SCAP/SREBP complex in the ER, thereby halting lipogenesis [204,206]. Similarly, DGAT1 blockade stops triglyceride synthesis, leaving excess free fatty acids to flood mitochondria via CPT1A for uncontrolled β-oxidation. This causes excessive ROS accumulation, mitochondrial damage, and apoptosis [205,207]. Under conditions of mitochondrial stress, peroxisomal α-oxidation and peroxisomal β-oxidation of very-long-chain fatty acids (VLCFAs) complement mitochondrial oxidation, with enzymes like medium-chain acyl-CoA dehydrogenase (MCAD) working to prevent lipid peroxidation-induced cell death in GSC niches [193]. Beyond preventing lipotoxic mitochondrial dysfunction, cytosolic lipid droplet (LD) accumulation serves as an essential cytoprotective barrier against ferroptosis. By selectively sequestering polyunsaturated fatty acids (PUFAs) into neutral triacylglycerols and cholesteryl esters, LDs reduce the abundance of free PUFAs available for membrane phospholipid incorporation, thereby neutralizing toxic lipid reactive oxygen species (ROS) accumulation and ferroptotic cell death [205]. Furthermore, this dynamic lipid buffering confers substantial chemoresistance against alkylating agents such as TMZ by mitigating drug-induced oxidative stress [189,205].

In addition to ferroptosis, inflammatory forms of regulated cell death, particularly gasdermin-mediated pyroptosis, may also influence glioma biology. Members of the gasdermin (GSDM) family can form membrane pores following proteolytic activation, promoting inflammatory cell lysis and the release of intracellular mediators. Emerging evidence suggests that dysregulated GSDM expression may contribute to glioma progression, immune microenvironment remodeling, and therapeutic responses [205].

### 3.4. The Microenvironment

#### 3.4.1. The Synaptic Interface

**Figure 1:**
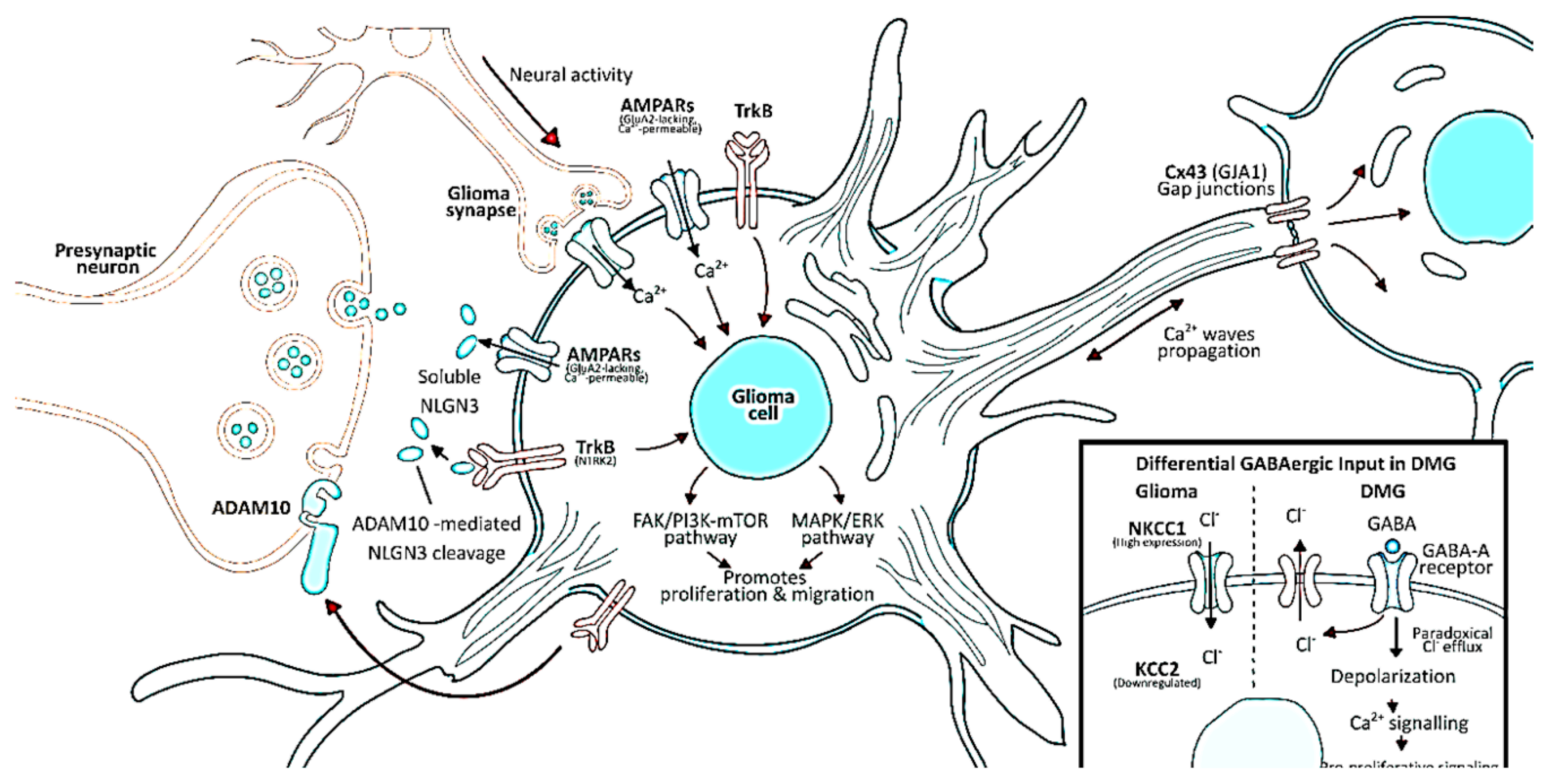
Synaptic integration and network interception by glioma microtubes.

Glioma progression relies on active integration into healthy central nervous system circuitry, establishing functional, electrochemical synapses with presynaptic neurons [208,209]. This direct coupling is mediated by calcium-permeable AMPARs lacking the post-transcriptionally edited GluA2 subunit, localized along TMs interconnected by connexin-43 (GJA1) [175,209,210]. Upon neuronal excitation, glutamate binding triggers massive Ca^2+^ influx and membrane depolarization, directly stimulating mitotic activity and invasion [211,212]. This stimulatory axis is sustained by bidirectional signaling: active neurons and oligodendrocyte precursor cells (OPCs) cleave and secrete the synaptic organizer neuroligin-3 (NLGN3) via vesicular ADAM10 sheddase, initiating focal adhesion kinase (FAK) phosphorylation upstream of the PI3K-mTOR, SRC, and SHC-RAS-RAF-MEK-ERK signaling cascades [213]. Simultaneously, neuronal activity induces brain-derived neurotrophic factor (BDNF) release, which binds to glioma-expressed TrkB (NTRK2) receptors, driving the trafficking and insertion of CP-AMPARs into the postsynaptic membrane in a feed-forward, long-term potentiation (LTP)-like mechanism (Figure 1) [211,214]. The local microenvironment is further modulated by extracellular tumor acidification (pH 5.5 to 3.4), which activates neuronal acid-sensing ion channel 1a (Asic1a), boosting the amplitude of excitatory postsynaptic currents to accelerate progression [215,216].

Remarkably, in H3K27M-altered DMGs, this hijacking extends to inhibitory circuits [34]. DMG cells express GABA_A_ receptor subunits and gephyrin postsynaptic scaffolding colocalized with presynaptic vesicular GABA transporter (VGAT) puncta [34]. Because of high expression of the chloride importer NKCC1 and silencing of the exporter KCC2, DMG cells maintain pathologically high intracellular chloride concentration [34]. Consequently, GABA receptor activation triggers a depolarizing Cl^−^ efflux that promotes tumor proliferation [217,218,219,220,221]. Clinically, while the AMPA antagonist perampanel halts growth and limits network hyperexcitability, GABA-enhancing benzodiazepines like lorazepam paradoxically accelerate DMG growth and shorten survival [34,222].

#### 3.4.2. Myeloid-Mediated Immunosuppression and Cellular Reprogramming

The TME of GBM represents an immunologically “cold” niche, dominated by TAMs that can comprise up to 50% of the total tumor mass [223,224,225]. This compartment displays a distinct spatial and ontogenetic structure: brain-resident microglia localize primarily at the peritumoral margin with pro-inflammatory interferon signatures, whereas bone marrow-derived macrophages (BMDMs), recruited via CCL2/CCR2, CX3CL1/CX3CR1, and CXCL12/CXCR4 axes, accumulate in the hypoxic core [223,224,226]. Under the influence of tumor-derived CSF-1, IL-10, and TGF-β, BMDMs polarize into an alternative CD163^+^CD206^+^ M2-like state, suppressing CD8^+^ T cells [223,226,227]. In mesenchymal GBM, a specialized subset of CD163^+^HMOX1^+^ microglia along with MARCO^+^ macrophages actively deplete T cells by emitting IL-10 [228].

Additionally, single-cell analysis reveals a highly suppressive, EGFR-amplified subpopulation characterized by high expression of secreted phosphoprotein 1 (SPP1/Osteopontin), termed TAM-SPP1 [229,230]. TAM-derived SPP1 interacts with CD44 and integrin receptors on malignant cells, driving a transition to a mesenchymal-like state and establishing an immunoexcluded fibrotic niche [230,231]. Intracellularly, SPP1 suppresses antitumor immunity by disrupting the ROS-mediated cGAS-STING/STAT1 signaling cascade, thereby silencing the transcription of chemokines CXCL9 and CXCL10, which drives CD8+ T-cell exclusion [232]. Concurrently, these TAMs exhibit a unique “neural growth” profile. Via SPP1, they activate neuronal mTORC2 signaling, driving active intratumoral axonal outgrowth and physical nerve infiltration [233]. This microenvironmental heterogeneity produces distinct therapeutic sensitivities: while proneural PDGFB-driven gliomas respond to TAM depletion via CSF1R inhibition (e.g., PLX3397), mesenchymal RAS-driven tumors resist due to TAM enrichment in pro-inflammatory and angiogenic signaling, a resistance bypassed only by co-targeting VEGF pathways [234].

### 3.5. Hypoxia, Vaso-Occlusion, and the Angiogenic Switch

Progression to CNS WHO grade 4 disease is histopathologically defined by central necrosis and microvascular hyperplasia, both of which are tightly regulated by localized tissue hypoxia. Rather than being a simple consequence of rapid metabolic demand, necrosis is actively initiated by vaso-occlusion and intravascular thrombosis [235,236]. Driven by PTEN loss and hypoxia, glioma cells upregulate Tissue Factor (TF/F3), the primary initiator of coagulation, creating a prothrombotic environment that precipitates extensive thrombus formation within tumor blood vessels [237,238]. Concurrently, upregulated angiopoietin-2 (ANGPT2) triggers endothelial cell apoptosis, initiating vessel regression [235,239].

**Figure 2:**
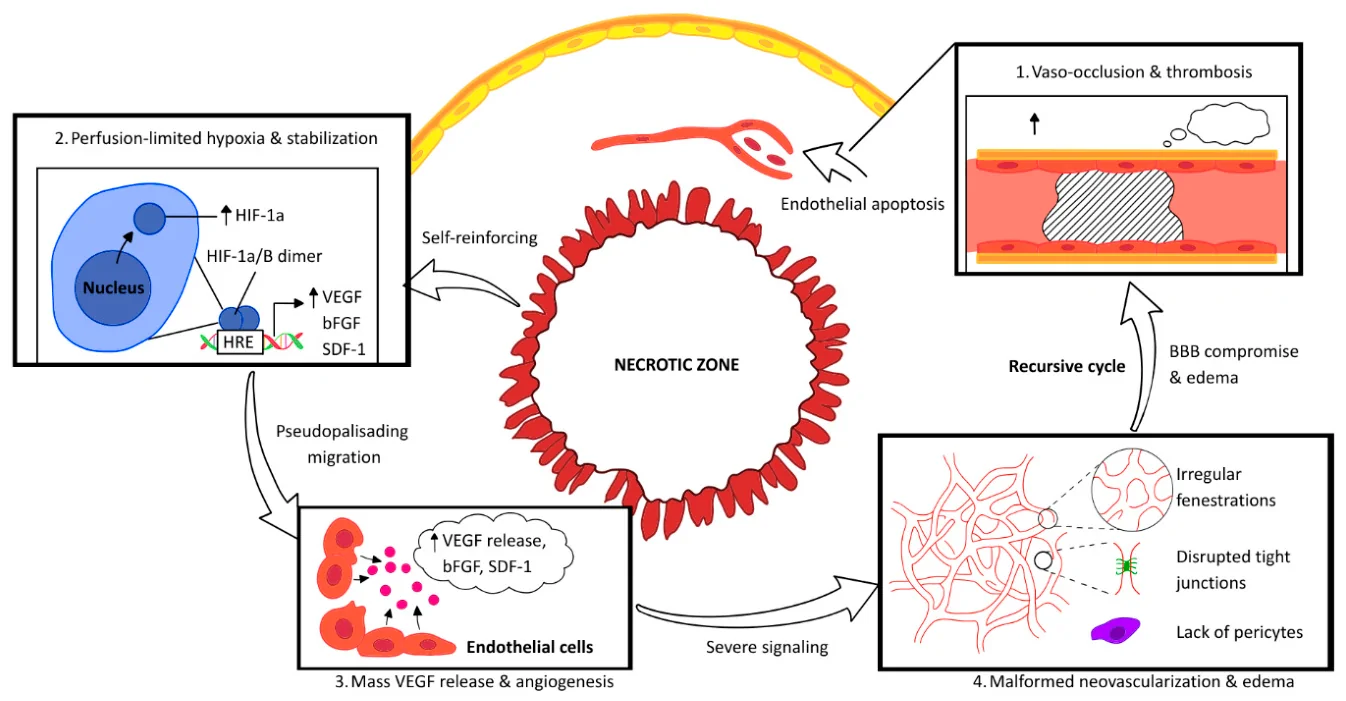
The hypoxic-angiogenic vicious cycle.

This perfusion-limited hypoxia stabilizes the transcription factor HIF-1α [240]. Once stabilized, HIF-1α translocates to the nucleus, dimerizes with HIF-1β, and binds to hypoxia-response elements (HREs) to transcriptionally activate its target genes, including VEGF, basic fibroblast growth factor (bFGF), and stromal cell-derived factor-1 (SDF-1) [241,242,243]. This severe localized signal provokes “pseudopalisading”, the active migration of hypoxic tumor cells away from the thrombosed, necrotic center. The massive release of VEGF by these migrating pseudopalisades induces microvascular hyperplasia [235]. However, the resulting vessels are structurally chaotic and immature, characterized by irregular fenestrations, disrupted tight junctions, and a lack of pericyte coverage [244]. This malformed architecture causes massive fluid extravasation, BBB compromise, and severe vasogenic edema, while failing to alleviate tissue hypoxia [245]. Consequently, a chronic, self-reinforcing hypoxic-angiogenic cycle is established, which protects GSCs in their perinecrotic niches and severely restricts the delivery of therapeutic agents (Figure 2, Table 2) [246].

### 3.6. Clonal Evolution and Cellular Plasticity

#### 3.6.1. Spatial and Temporal Heterogeneity

Malignant cells within IDH-wildtype GBM reject rigid, hierarchical models of development, adopting instead a highly plastic framework structured around diverse, interconvertible transcriptomic states [18,19,247]. Landmark single-cell transcriptomic profiling delineated four primary cellular states that recapitulate neuro-developmental programs: AC-like, OPC-like, NPC-like, and MES-like [17,18,248]. Rather than being stochastic, these states are biased by somatic copy-number alterations: copy-number amplifications of the EGFR locus promote the AC-like state, PDGFRA alterations favor the OPC-like lineage, CDK4 amplifications enrich the NPC-like state, and NF1 loss drives the MES-like program [18,248,249].

The dynamic spectrum of GBM states is far more elaborate than previously realized. Large-scale single-nucleus RNA sequencing (snRNA-seq) performed by the Cellular Analysis of Resistance and Evolution (CARE) consortium on 121 specimens from 59 patients expanded this classic model, revealing novel, highly specialized malignant cell states [18,250]. Among these, the glial progenitor cell-like (GPC-like) state occupies a central, highly proliferative position (comprising approximately 12.5% cycling cells) at the intersection of astrocytic, neuronal, and mesenchymal differentiation trajectories [251]. Characterized by the expression of tyrosine kinase genes EGFR and ALK, alongside core transcription factors such as MEIS1, MEOX2, ETV1, ELOVL2, and KLHDC8, GPC-like cells act as high-plasticity engines of glioma renewal [252,253,254].

Conversely, the neuronal-like (NEU-like) state demonstrates significant transcriptional overlap with mature excitatory neurons, expressing synaptic and axonal markers such as SYN3, SYT1, NRG1, NRG3, NRXN3, and CNTNAP2 [251]. Because NEU-like cells possess complex cellular morphologies, they are heavily depleted during standard enzymatic tumor dissociation protocols, remaining virtually invisible to traditional single-cell sequencing while being clearly resolved via single-nucleus platforms [18]. Additionally, a rare, lineage-restricted cilia-like state (comprising approximately 1.6% of the tumor population) has been identified, where a subset of AC-like cells activates a specialized axonemal program expressing DNAH and CFAP gene families [251]. Finally, a distinct cellular program, identified as the stress state, coalesces two discrete stress-associated signatures (stress1 and stress2) that are characterized by heat shock and unfolded protein responses linked to highly disordered local DNA methylation [251,255].

To contextualize these findings, these diverse cellular programs do not function in isolation. Rather, after accounting for individual state abundances, three highly consistent, state-independent consensus baseline profiles govern intertumor heterogeneity: neuronal, glial, and extracellular matrix (ECM) [251]. These distinct baseline programs co-evolve alongside nonmalignant stroma, coordinating complex ligand-receptor loops that organize GBMs into three stereotypic, clinically resilient ecosystems [251].

Crucially, highly resistant “hybrid” populations exist at the interface of these cellular states, challenging the traditional view that extreme transcriptomic states are mutually exclusive [256]. High-resolution tracking utilizing synthetic fluorescent reporter systems has identified a distinct proneural-mesenchymal (PM) hybrid cell population concentrated primarily within the non-hypoxic tumor core, rather than being restricted to hypoxic niches [256,257]. Representing the top 10% of double-reporter-positive cells, these hybrid cells are highly proliferative, possess highly open chromatin landscapes, and are enriched after standard-of-care chemoradiotherapy [256]. At the molecular level, these cells exhibit c-Myc activation and an extensively upregulated nucleocytoplasmic transport machinery [256]. This transport network relies on nuclear export proteins and importins, specifically KPNB1, IPO5, and KPNA2, which facilitate rapid transcription factor trafficking to maintain plastic, intermediate states [256,258,259]. Consequently, the targeting of this nucleocytoplasmic shuttle represents an actionable therapeutic vulnerability to suppress adaptive cell plasticity and re-sensitize resistant populations to DNA-damaging agents [256].

#### 3.6.2. Glioma Stem Cells Niche

The persistence of glioma GSCs is governed by two primary microanatomical niches: the perivascular niche (PVN) and the perinecrotic niche [260,261,262,263]. In the PVN, GSCs reside adjacent to disorganized blood vessels undergoing rapid microvascular proliferation (MVP) [264,265,266]. This dysfunctional endothelial structure fractures the BBB, recruiting immunosuppressive TAMs, myeloid-derived suppressor cells (MDSCs), and microglia [267]. GSC stemness and survival within this niche are sustained through extensive ligand-receptor loops involving endothelial- and pericyte-derived growth factors, specifically VEGF, EGF, FGF, ANG2, TGFβ, ephrinB2, and PDGF [268,269,270,271,272,273,274,275].

As tumors outgrow their local blood supply, severe oxygen deprivation triggers the hypoxic and perinecrotic niches [276]. Driven by hypoxia-inducible factors HIF-1α and HIF-2α, hypoxia serves as a primary force of therapy resistance by reshaping DNA repair fidelity and transporter kinetics [277,278]. Long-term hypoxia downregulates homologous recombination, nucleotide excision repair, and mismatch repair pathways, leading to genomic instability while preventing the oxidation-mediated free radical damage necessary for chemotherapy- and radiation-induced apoptosis [276]. Instead, hypoxic GSCs tolerate massive DNA damage through translesion DNA synthesis facilitated by proliferating cell nuclear antigen-associated factor (PAF) [279]. Simultaneously, multidrug resistance is enforced through HIF-mediated transporter expression. Cyclic hypoxia drives HIF-1α-dependent transcription of ABCB1, while HIF-2α upregulates ABCG2 via the stemness transcription factor OCT4 [280,281,282]. CD133-positive GSC populations residing in these perinecrotic zones exhibit marked resistance to standard alkylating stressors (e.g., TMZ) through elevated expression of ABCB5 [283].

Microenvironmental stress and radiotherapy actively drive the PM transition (PMT), a phenotypic switch mirroring carcinoma epithelial–mesenchymal transition [284]. This state transition is coordinated by the master transcription factors STAT3 and C/EBPβ, while TAZ acts as an independent regulatory bypass [285,286,287]. In low-grade gliomas, the TAZ promoter remains hypermethylated, but its demethylation in grade IV GBM unleashes potent mesenchymal reprogramming [286,288]. Further reinforcing this state, CXCR4 signaling drives PMT by orchestrating the upregulation of STAT3, C/EBPβ, and TAZ regulons, shortening the OS in GBM patients [286,289].

The apex regulator of PMT, however, is Fos-like antigen 1 (FOSL1), a component of the AP1 complex highly expressed in mesenchymal GSCs [290]. FOSL1 transcriptionally activates the E2 SUMO-conjugating enzyme UBC9 [290]. This enzymatic induction promotes the SUMOylation of the deubiquitinating enzyme CYLD, which subsequently triggers K63-linked polyubiquitination of nuclear factor κB (NF-κB) intermediaries [290]. This cascade unleashes robust NF-κB transcription, inducing the mesenchymal phenotype and protecting GSCs from radiation-induced apoptosis [290].

#### 3.6.3. Treatment-Induced Bottlenecks and Hypermutator Phenotypes

The standard-of-care regimen, combining surgical resection, ionizing radiation, and TMZ chemotherapy, imposes a massive evolutionary bottleneck that selects for highly aggressive, therapy-resistant clones [291]. TMZ exerts its cytotoxicity through the alkylation of DNA, generating adducts such as N7-methylguanine (70%), N3-methyladenine (9%), and highly mutagenic O^6^-methylguanine (O^6^-meG, 5%) [292]. In tumors featuring epigenetic silencing of the direct-reversal enzyme MGMT, these O^6^-meG lesions mispair with thymine. In mismatch repair (MMR)-proficient cells, this mispair triggers recurrent, futile cycles of MMR that generate double-strand DNA breaks, leading to G2/M cell cycle arrest and apoptosis [293,294].

This cytotoxic pressure, however, drives a severe evolutionary paradox. Under prolonged alkylator exposure, cells frequently acquire defects in the mismatch repair machinery, primarily through somatic mutations or inactivation of the MSH6 gene (such as the recurrent Thr1219Ile mutation) [291]. Deprived of functional MSH6, cells escape O^6^-meG:T recognition, surviving the alkylating stress but initiating a hypermutator phenotype [291,295]. This hypermutated state, clinically characterized by the somatic mutational signature SBS11 (CpC transitions), drives extensive spatial diversification, rapid radiological progression, and resistance to salvage therapies [295].

At the tissue level, longitudinal profiling by the Glioma Longitudinal Analysis (GLASS) Consortium has mapped these evolutionary trajectories in matched primary-recurrent pairs [296,297,298]. Standard chemoradiation triggers cellular senescence, and the resulting senescence-associated secretory phenotype (SASP) serves as a major driver of post-treatment dynamics [298]. Crucially, longitudinal SASP dynamics are highly subtype-specific. Mesenchymal tumors show rapid clearance of SASP at recurrence (ΔSASP = −0.83, *p* = 0.0001), driven by the robust surveillance of invading M2 tumor-associated macrophages. In contrast, classical tumors maintain highly stable SASP levels (*p* = 0.86) [298].

This failure to clear the SASP creates a high-risk group of “SASP Accumulators”. A rising SASP trajectory strongly predicts PMT at recurrence, with each unit increase in ΔSASP yielding five-fold higher odds of PMT (OR = 5.06, 95% CI: 2.30–11.18, *p* < 0.0001). This transition is strongly linked to immunosuppressive M2 macrophage infiltration (rho = 0.65, *p* < 0.0001) and severely shortened overall survival (HR = 2.48, *p* = 0.0001). Conversely, “SASP Clearers” are nearly completely protected from undergoing PMT (transition rate of only 2.4% versus 27.3% in Accumulators, *p* = 0.0008) [298].

While canonical inflammatory SASP factors such as IL1A, IL1B, and MMP9 decrease significantly at recurrence, hepatocyte growth factor (HGF) represents a striking exception. HGF is uniquely upregulated in recurrent tumors (Δ = +0.23, *p* = 0.016), serving as a crucial, senescence-escape signaling bypass that fuels aggressive post-bottleneck clonal expansion and recurrence [298].

These genomic observations are further reinforced by the CARE consortium’s longitudinal profiling of paired primary-recurrent GBMs. Their multi-layered single-cell transcriptomic analyses revealed that there is no single, uniform longitudinal trajectory of malignant cell states across GBM cohorts. Instead, the predominant malignant state shifted between matched pairs in a highly patient-specific manner, yet tracked dynamically with co-evolving changes in the nonmalignant TME [250,251]. Remarkably, those patients who developed a glio-neural TME at recurrence (characterized by a recovery of reactive astrocytes and neuronal cell-type signatures) experienced a significantly prolonged survival after their second surgery compared to patients returning with ECM or glial-rich immunosuppressive ecosystems (median OS of 19 months versus 8 months, *p* = 0.012, log-rank test) [250]. These findings demonstrate that GBM progression is not a purely cell-autonomous trajectory, but is rather a co-evolutionary struggle shaped by spatial microenvironments, microanatomical niches, and treatment-induced selection pressures [250,299,300].

#### 3.6.4. Translational Frontiers and Targeted Clinical Strategies

Translating these complex mechanistic insights into tangible clinical survival benefits represents the defining challenge of modern neuro-oncology. Historically, monotherapeutic targeted trials (e.g., against EGFRvIII, VEGF, or single tyrosine kinases) uniformly failed due to target loss, redundant collateral cascades, and compensatory transcriptomic plasticity [6,36]. Consequently, contemporary clinical trial designs are increasingly shifting toward combinatorial and microenvironment-disrupting paradigms.

Directly targeting the neuro-glioma electrochemical interface has emerged as a particularly promising frontier. Based on preclinical demonstrations that AMPAR-mediated synaptic depolarization directly drives tumor proliferation, the non-competitive AMPA receptor antagonist perampanel is currently under active clinical investigation (e.g., NCT04566380) to evaluate its dual capacity to suppress glioma-associated epileptiform hyperexcitability and halt tumor progression [212,222]. Similarly, the targeting of downstream neuro-developmental ligands using the metalloproteinase inhibitor INCB7839 to block ADAM10-mediated cleavage of neuroligin-3 (NLGN3) represents an innovative strategy to sever neuronal growth inputs [213].

In the oncohistone space, diffuse midline gliomas harboring H3 K27 alterations, long considered refractory to systemic therapy, are demonstrating unprecedented clinical responses to the small-molecule imipridone ONC201 (dordaviprone) [300]. Acting as a selective antagonist of dopamine receptor D2 (DRD2) and an allosteric agonist of the mitochondrial protease ClpP, ONC201 disrupts mitochondrial respiration, degrades PRC2 components, and selectively induces apoptosis in H3K27M-mutant cells, showing durable radiographic responses and significant survival extension in multi-center clinical evaluations [300]. Concurrently, the breakthrough success of the dual mIDH1/2 inhibitor vorasidenib in the phase III INDIGO trial (NCT04164901) has validated early epigenetic intervention in lower-grade IDH-mutant tumors, delaying malignant grade progression and delaying the requirement for genotoxic chemoradiotherapy [79].

Ultimately, overcoming GBM’s evolutionary resilience will require multi-arm, platform trials (such as the AGILE initiative, NCT03970447) that dynamically evaluate combinations targeting core metabolic engines (e.g., lipid droplet and glutamine metabolism), immune checkpoints, and neuro-synaptic circuitry simultaneously, rather than static single-target blockades [300].

Despite the transformative mechanistic insights outlined in this review, several critical limitations of the current literature must be acknowledged. First, a substantial proportion of functional and metabolic findings remains derived from preclinical murine models, two-dimensional cell cultures, and serum-free gliomaspheres, which fail to fully recapitulate the intricate, multi-cellular human neuro-vascular-immune milieu and native architectural biomechanics [299]. Second, while single-cell and single-nucleus transcriptomic technologies have resolved profound cellular heterogeneity, they provide static snapshots of highly dynamic transcriptional trajectories, potentially oversimplifying transient intermediate states [18,250]. Finally, longitudinal profiling of primary-recurrent pairs remains constrained by spatial sampling bias during surgical cytoreduction, where localized tissue specimens may not fully capture distant, infiltrating subclonal reservoirs [68,300]. Addressing these translational gaps through longitudinally tracked spatial multi-omics, patient-derived organoid-immune co-cultures, and clinically integrated platform trials will be essential to translate molecular targets into durable clinical therapies [299,300].

## 4. Conclusions

The molecular exploration of GBM reveals that its profound clinical lethality is not a product of random cellular chaos, but rather the result of a highly coordinated, resilient network ecosystem. This persistent therapeutic stagnation, marked by an unchanged standard of care and an overall survival rate that has plateaued for over twenty years, stems from an incomplete appreciation of the tumor’s biological complexity. Rather than acting as a static, uniform mass of proliferating cells, GBM operates as a non-genetically plastic, interconnected, communicating cellular network. It dynamically restructures its transcriptomic and metabolic identity in direct response to microenvironmental stress and cytotoxic pressure. The taxonomic shift introduced by the WHO CNS5 classification system successfully mirrors this biological truth, anchoring diagnosis within explicit molecular alterations such as IDH-wildtype status, TERT promoter mutations, and EGFR amplifications. This ensures that future clinical interventions are aligned with the tumor’s intrinsic genetic reality rather than its superficial microscopic shape.

A central pillar of GBM’s survival strategy is its rejection of rigid, unidirectional cellular hierarchies. Cells shift fluidly across a developmental continuum, transitioning between astrocyte-like, mesenchymal-like, neural progenitor-like, and oligodendrocyte progenitor-like states. Recent single-nucleus sequencing has expanded this paradigm by uncovering highly proliferative hybrid populations, including PM hybrid states. These hybrid cells act as critical cellular substrates of recurrence. They exploit specialized nucleocytoplasmic transport machinery, utilizing importins and exportins like KPNB1 and IPO5 to maintain intermediate, highly adaptable phenotypes that effortlessly evade single-pathway targeted blockades. This structural flexibility is further supported by an intricately rewired epigenome. Whether initiated by the neomorphic production of the oncometabolite 2-hydroxyglutarate establishing the G-CIMP phenotype, followed by subsequent progressive demethylation during G-CIMP-low transitions, or governed by the global depletion of H3K27me3 marks by H3K27M oncohistones, chromatin remodeling acts as a master switch that opens hidden enhancer landscapes and locks cells into permanent, self-renewing developmental loops.

Crucially, the tumor’s aggressively invasive nature is fed by an active, functional neuro-gliomal integration into the host brain’s functional circuitry. By forming active glutamatergic synapses along TMs and paradoxically transforming inhibitory GABAergic signals into depolarizing stimuli, malignant cells capture neuronal excitation to fuel their own proliferation. This electrical hijacking works in tandem with a plastic metabolic engine that balances the Warburg effect with oxidative phosphorylation, scavenges the brain’s amino acid reservoirs, and sequesters neutral lipids into lipid droplets to avoid lipotoxicity. Surrounded by an immunologically cold microenvironment, where SPP1+ bone marrow-derived macrophages exclude CD8^+^ T cells and tissue factor-induced vaso-occlusions fuel a self-reinforcing hypoxic-angiogenic cycle, the tumor constructs a highly protective, immunosuppressive microenvironmental niche.

Ultimately, conventional therapies impose an intense evolutionary bottleneck that ironically shapes the trajectory of recurrence. Under the genotoxic stress of temozolomide, the loss of mismatch repair enzymes like MSH6 triggers a hypermutator phenotype, accelerating spatial diversification. Furthermore, longitudinal dynamics reveal that a patient’s survival depends heavily on how the microenvironment handles cellular senescence. “SASP Accumulators” fail to clear senescent signaling, driving a rapid proneural-to-mesenchymal transition (OR = 5.06) and recruiting immunosuppressive M2 macrophages (rho = 0.65). Conversely, patients who clear the SASP or establish a glio-neural microenvironment at recurrence experience a significantly extended overall survival (median OS = 19 months versus 8 months, *p* = 0.012). Therefore, improving therapeutic outcomes in GBM will likely require a shift away from static monotherapies toward rationally designed, multi-targeted combination regimens. Future clinical trials must integrate agents that disrupt synaptic hijacking (e.g., AMPAR or ADAM10 inhibitors) and metabolic sanctuaries with strategies that inhibit cellular plasticity shuttles (e.g., nucleocytoplasmic transport) and dismantle immunosuppressive myeloid niches, thereby trapping this dynamic tumor network in an evolutionarily unviable state.

## Figures and Tables

**Table 1:** Comparison of molecular hallmarks in IDH-Mutant Diffuse Astrocytoma and IDH-Wildtype Glioblastoma.

Molecular Hallmarks	IDH-Mutant Diffuse Astrocytoma (Progression to Grade IV)	IDH-Wildtype Glioblastoma (De Novo Grade IV)	Sources
Median age of onset	~40 years	~62 years	[51,78,79]
Cell of origin	Neural stem cells; develops from low-grade precursors	Neural stem cells (NSCs) residing in the subventricular zone (SVZ), or oligodendrocyte precursor cells (OPCs); develops de novo	[38,44,51,62,80,81]
Key initiating events	IDH1 or IDH2 mutations; TP53 mutations; ATRX loss	EGFR mutations; TERT promoter mutations; chromosome +7/−10	[46,64,71,82,83]
Progression drivers	Homozygous CDKN2A/B deletion; LOH of 10q; global loss of G-CIMP methylation	Intrinsic pathway activation (PTEN loss, MDM2 amplification, CDKN2A deletion)	[37,38,51,62,64,77]
Epigenetic landscape	Hypermethylated G-CIMP-high state, which may transition to C-CIMP-low during progression	Unmethylated or locus-specific promoter methylation	[77,81,82,83,84]
Prognosis	Initially favourable; median OS drops to 3–5 years upon CDKN2A/B homozygous loss	Highly aggressive; median OS 15–16 months	[44,53,55,83,85,86]

**Table 2:** Summary of the characteristics of glioma microenvironment niches.

Microenvironmental Segment	Dominant Cellular Elements	Primary Driver Signaling Cascades	Pathophysiological Consequences
Synaptic niche	Presynaptic neurons, postsynaptic glioma cells, TMs	FAK/PI3K-mTOR, BDNF-TrkB LTP, GABA depolarization via NKCC1	Electrically active networks, accelerated tumor cell proliferation and invasion
Immunosuppressive myeloid niche	Recruited BMDMs (CD163+CD206+), resident microglia	SPP1-CD44/Integrin axes, CCL2-CCR2 recruitment, ROS-cGAS-STING silencing	CD8+ T-cell exclusion, MES-like state transitions, tumor innervation
Necrotic and vascular niche	Pseudopalisading tumor cells, chaotic endothelial cells	TF/F3 coagulation, HIF-1α-VEGF-A/ANGPT2 axis	Vaso-occlusion, BBB failure, vasogenic edema, drug delivery failure

## Data Availability

No new data were created or analyzed in this study. Data sharing is not applicable to this article.
